# The PD-L1–mTOR intersection in tumor migration and invasion

**DOI:** 10.1007/s11033-026-12982-6

**Published:** 2026-10-23

**Authors:** Rafael Palavro, Thales Thor Ramos Previato, Douglas Bottega Pazzin, Júlia Budelon Gonçalves, Marina Ottmann Boff, Daniel Marinowic, Jaderson Costa da Costa, Thamiris Becker Scheffel

**Affiliations:** 1 Pontifical Catholic University of Rio Grande do Sul, Porto Alegre, RS Brazil; 2 Brain Institute of Rio Grande do Sul, Pontifical Catholic University of Rio Grande do Sul, Porto Alegre, RS Brazil; 3 Department of Pharmacology, Institute of Basic Health Sciences, Federal University of Rio Grande do Sul, Porto, Alegre, RS, Brazil

**Keywords:** Programmed death-ligand 1, Mechanistic target of rapamycin, Epithelial-mesenchymal transition, Tumor cell invasion

## Abstract

Programmed death-ligand 1 (PD-L1) has emerged as an important player in intrinsic tumor cell biology besides its canonical immune checkpoint function. Cumulative experimental data points to a role for PD-L1 in promoting and maintaining an invasive cell phenotype within different cancer cells through its interaction with mechanistic target of rapamycin (mTOR) signaling. In this review, we summarize the main experimental data pointing to the PD-L1/mTOR interaction in tumor cell invasion capacity both in vitro and in vivo. In this crosstalk, PD-L1 expression is induced by IFN-γ/STAT1 and IL-6/STAT3 pathways, as well as mTOR activity by its upstream effectors like EGFR and other tyrosine kinase receptor ligands. Interestingly, PD-L1 itself seems to play a role in mTOR activation. In many of these models, the PD-L1 importance in cell migration capacity is evidenced by two major observations: (1) its knockout impairs cell invasion capacity and, (2) its upregulation is associated with increased cell migration and both the induction and maintenance of epithelial-mesenchymal transition (EMT) phenotype markers, such as increased N-cadherin and ZEB1 and decreased E-cadherin. We also summarize the main clinical trials combining mTOR pathway inhibitors with anti-PD-L1 antibodies in different cancer types.

## Introduction

Programmed death-ligand 1 (PD-L1), also referred to as *CD274* or *B7-H1*, has emerged as a central integrator of immune evasion, metabolic reprogramming, and tumor cell plasticity. It is a transmembrane glycoprotein of the B7 family within the immunoglobulin superfamily [1], broadly expressed in lymphoid and non-lymphoid tissues, including T and B cells, macrophages, and dendritic cells [2]. Structurally, PD-L1 comprises immunoglobulin variable (IgV)-like and immunoglobulin constant (IgC)-like extracellular domains responsible for receptor binding and protein stability [3, 4]. Beyond its membrane-associated form, PD-L1 also displays cytoplasmic, nuclear, and soluble isoforms, highlighting its dynamic regulation and functional versatility [5, 6].

The programmed cell death protein 1 (PD-1), also known as *PDCD1* or *CD279*, is expressed on activated lymphocytes and other immune subsets [7]. Upon ligand binding, phosphorylation of the immunoreceptor tyrosine-based inhibitory motif (ITIM) and immunoreceptor tyrosine-based switch motif (ITSM) promotes recruitment of the Src homology region 2 domain-containing phosphatases SHP-1 and SHP-2, leading to inhibition of T cell receptor (TCR) signaling and attenuation of immune activation [8].

The PD-1/PD-L1 axis is a key immune checkpoint in the adaptive immune response, essential for maintaining immune homeostasis. Under physiological conditions, the interaction between PD-1 and its ligands (PD-L1 or PD-L2) limits excessive T lymphocyte activation [7]. This interaction suppresses TCR-mediated signaling, resulting in reduced proliferation, cytokine production, and cytotoxic activity of T lymphocytes [9, 10]. Thus, while this pathway limits excessive immune activation, it can also constrain effective immune responses in diseases through its immune checkpoint effect.

The dysregulation of this axis is involved in different pathological contexts. In autoimmune diseases, impaired PD-1/PD-L1 signaling undermines proper immune tolerance [11, 12]. In cancer, this axis is frequently co-opted through PD-L1 upregulation, enabling tumor cells to evade immune surveillance and establish an immunosuppressive microenvironment. Several tumor types, including lung cancer, melanoma, glioma, and breast cancer, exhibit elevated levels of PD-L1, which are often associated with poorer clinical prognosis [8–10, 13]. Besides tumor-intrinsic upregulation, the expression of PD-L1 can be due to an adaptive immune resistance, in which the presence of immune infiltrate stimulates PD-L1 upregulation in cancer cells [14]. In this context, it is important to note that PD-L1 expression in cancer cells is not necessarily a feature of intrinsic tumor biology or reduced immune response. It can rather be a consequence of cytokines secreted from a pre-active immune response and lymphocyte infiltrate [15, 16].

The tumor microenvironment (TME) represents a dynamic niche that integrates inflammatory, metabolic, and cellular stress signals to support tumor progression. In this context, proinflammatory cytokines such as interferon-gamma (IFN-γ), tumor necrosis factor-alpha (TNF-α), and interleukin-6 (IL-6) are key inducers of PD-L1 expression in tumor cells, reinforcing immunosuppression and promoting adaptive resistance mechanisms [17, 18]. These signals simultaneously drive metabolic rewiring pathways, establishing a mechanistic link between immune evasion and metabolic adaptation within the TME.

Beyond its canonical immunosuppressive function, PD-L1 exerts tumor-intrinsic regulatory roles associated with stemness and invasive phenotypes. Tumor cell invasion is closely linked to epithelial-mesenchymal transition (EMT), a process characterized by increased N-cadherin and vimentin, and decreased E-cadherin expression, leading to increased migration and invasion. This phenotypic reprogramming is orchestrated by transcription factors such as Snail family transcriptional repressor 1 (SNAIL1), Snail family transcriptional repressor 2 (SNAIL2, also known as SLUG), and zinc finger E-box-binding homeobox 1 (ZEB1) [19]. Importantly, emerging evidence indicates that PD-L1 actively participates in the regulation of EMT-associated transcriptional processes, linking immune checkpoint signaling to tumor cell plasticity.

In the immunometabolism context, PD-L1 exhibits dual functionality: extrinsically, it suppresses cytotoxic T lymphocytes activity, reducing their glucose uptake and cytokine production, thereby favoring resource utilization by the tumor cells; intrinsically, it activates the phosphoinositide 3-kinase (PI3K)/protein kinase B (AKT)/mechanistic target of rapamycin (mTOR) signaling axis, promoting glycolysis, anabolic metabolism, and resistance to metabolic stress. Collectively, these coordinated mechanisms enhance tumor metabolic fitness and sustain tumor progression [20–22].

mTOR is a central serine/threonine kinase that regulates cell growth, metabolism, proliferation, and survival by integrating environmental, metabolic, and genetic signals that maintain cellular homeostasis [23]. mTOR functions through three distinct complexes: mTOR complex 1 (mTORC1), mTOR complex 2 (mTORC2), and the more recently described mTOR complex 3 (mTORC3), the latter primarily identified in tumor contexts and characterized by its ability to sustain mTOR kinase activity even under rapamycin inhibition [24]. While mTORC1 is activated by amino acids, growth factors, and cellular energy levels, promoting protein synthesis, lipogenesis, and mitochondrial biogenesis, mTORC2 predominantly regulates cell proliferation, survival, glucose metabolism, and cytoskeletal organization [25], also contributing to the maintenance of cancer stem cell (CSC) self-renewal properties [26].

Dysregulation of mTOR signaling is a hallmark of multiple pathologies, including cancer. In tumor cells, mTOR signaling is closely linked to the regulation of PD-L1 expression through the activation of oncogenic pathways such as PI3K/AKT/mTOR, rat sarcoma viral oncogene homolog (RAS), epidermal growth factor receptor (EGFR), mitogen-activated protein kinase kinase (MEK)/extracellular signal-regulated kinase (ERK), and p38 mitogen-activated protein kinase (MAPK), promoting immune evasion via increased PD-L1 expression [27]. In turn, PD-L1-driven signaling may further reinforce these pathways, suggesting the existence of a regulatory loop between metabolic signaling and immune checkpoint control.

Beyond its canonical function, PD-L1 has emerged as a key regulator of tumor-intrinsic processes, including EMT and the acquisition of invasive phenotypes. Notably, PD-L1 expression appears to be tightly controlled by mTOR signaling, highlighting a functional crosstalk between metabolic regulation and immune checkpoint pathways. In this review, we summarize the experimental evidence supporting the non-canonical roles of PD-L1, with emphasis on EMT and tumor invasion, and discuss how the mTOR integrates extracellular and intracellular signals to modulate PD-L1 expression and drive cancer progression, positioning the PD-L1/mTOR axis as a central node linking immune escape, metabolic fitness, and tumor invasiveness.

## PD-L1 beyond immune evasion

Over the past decade, PD-L1 has emerged as a central regulator of tumor-intrinsic signaling, extending beyond immune evasion. Accumulating evidence demonstrates that PD-L1 drives cell-autonomous programs controlling metabolism, proliferation, and phenotypic plasticity across cancer types. These non-canonical functions converge on growth and metabolic pathways, particularly the PI3K/AKT/mTOR axis, positioning PD-L1 as a key node linking immune escape to tumor-intrinsic adaptation.

### Functions of PD-L1

At the functional level, PD-L1 signaling converges on tumor plasticity programs, including EMT and CSC maintenance, thereby linking immune evasion to invasion, metastasis, and therapeutic resistance.

Genetic or pharmacological suppression of PD-L1 reveals direct cell-intrinsic dependencies in tumor cells across multiple cancer types. In gastric cancer cells, PD-L1 knockdown significantly impaired cell proliferation, migration, invasion, cell-cycle progression, tumorigenicity, and susceptibility to cytokine-induced killer cell mediated cytotoxicity [28]. Similarly, in triple-negative breast cancer (TNBC), PD-L1 depletion reduced tumor cell proliferation and triggered apoptosis through both intrinsic and extrinsic apoptotic pathways [29]. In murine sarcoma models, tumor-specific PD-L1 blockade disrupted tumor growth and attenuated glycolytic metabolism via suppression of mTOR signaling [21]. Consistent with these findings, reduced PD-L1 expression in ovarian cancer and melanoma cells was linked with diminished proliferative capacity in vitro compared with control cells [30].

PD-L1 functions as an intracellular signaling molecule, exerting regulatory roles within the cytoplasm and nucleus that are independent of its immune role. In TNBC cells, cytoplasmic PD-L1 can bind and stabilize specific mRNA transcripts, thereby enhancing the expression of DNA damage response proteins [31]. In addition, perinuclear or nuclear PD-L1 has been shown to work with DNA-dependent protein kinase, leading to activation of the MEK/ERK signaling pathway, which cooperates with mTOR-driven anabolic and cytoskeletal functioning to sustain invasive phenotypes [32]. PD-L1 nuclear translocation is further modulated by interactions with vimentin, phosphorylated signal transducer and activator of transcription 3 (STAT3; pSTAT3-Y703), and karyopherin β1 (KPNB1), which facilitate nuclear import, whereas p300 acts as a negative regulator of this process. Once localized to the nucleus, PD-L1 can transcriptionally activate pro-tumorigenic genes, including growth arrest-specific 6 (*GAS6*), WAS/WASL-interacting protein (*WIP*), and catenin beta 1 (*CTNNB1*), through direct binding to specific promoter regions. These findings position nuclear PD-L1 as a transcriptional co-regulator that reprograms gene expression to favor tumor progression [33, 34].

Consistent with these findings, PD-L1 expression correlates with CSC-like phenotypes across multiple tumor types, commonly marked by high CD44 and/or CD133 expression [35–37]. Complementary RNA interference studies demonstrate that PD-L1 can directly regulate core stemness transcription factors, including octamer-binding transcription factor 4 (OCT4), Nanog homeobox (NANOG), and BMI1 proto-oncogene, polycomb ring finger (BMI1), reinforcing its functional involvement in CSC regulatory networks [38]. PD-L1 reciprocally promotes CSC maintenance by inducing OCT4 and NANOG expression through PI3K/AKT signaling, which feeds directly into mTOR-dependent control of growth, metabolism, and self-renewal [39]. Accordingly, PD-L1 depletion compromises CSC self-renewal both in vitro and in vivo, establishing PD-L1 as an active driver of stemness rather than a passive CSC marker [40].

PD-L1 can also actively promote EMT and invasive behavior through engagement of multiple signaling axes. In colorectal carcinoma, it induces a stem and mesenchymal state by interacting with high mobility group AT-hook 1 (HMGA1) and activating PI3K/AKT and MEK/ERK pathways [41]. Similarly, in glioblastoma (GBM) and other epithelial malignancies, PD-L1 overexpression reprograms EMT transcriptional networks, characterized by upregulation of N-cadherin, vimentin, SLUG, and β-catenin alongside downregulation of E-cadherin. These effects are mediated through ERK-dependent mechanisms that intersect with cytoskeletal remodeling and migratory programs [42]. The upregulation of PD-L1 can also enhance EMT by sterol regulatory element-binding protein 1 (SREBP-1) [43, 44].

In hypopharyngeal squamous cell carcinoma, PD-L1 knockdown reduced N-cadherin and vimentin expression in parallel with diminished AKT phosphorylation [45], whereas enforced PD-L1 expression augmented migration and invasion in nasopharyngeal carcinoma through activation of the PI3K/AKT pathway [46]. Furthermore, in EGFR-mutant non-small-cell lung cancer (NSCLC), PD-L1 was shown to promote migration and invasion by enhancing Smad3 phosphorylation and activating the transforming growth factor beta (TGF-β)/Smad signaling axis, concomitantly conferring resistance to the EGFR tyrosine kinase inhibitor (TKI) gefitinib [47].

PD-L1 has also been implicated in the promotion of tumor cell proliferation through phosphorylation of the mTORC1 substrate 70 kDa ribosomal protein S6 kinase (p70S6K) at Thr389, thereby inducing mTORC1 activity [30]. Mechanistically, the PI3K/AKT/β-catenin/WIP signaling cascade has emerged as a key mediator of PD-L1-driven EMT, wherein PD-L1 upregulates β-catenin, facilitates its binding to the WIP promoter, and induces WIP expression, ultimately modulating actin polymerization and cytoskeletal dynamics [48].

### Regulation of PD-L1

Extracellular inflammatory and intracellular oncogenic signaling pathways, which converge on tumor growth and metabolic control, regulate PD-L1 expression. Among extracellular signaling, IFN-γ is a principal inducer of PD-L1, along with pro-inflammatory cytokines such as interleukin 17 (IL-17), IL-6, TNF-α, TGF-β1, and hypoxia-associated signaling mediated by hypoxia-inducible factor 1 alpha (HIF-1α) [49–52] .At the intracellular level, oncogenic signaling pathways including activation of mTORC1 consequent to phosphatase and tensin homolog (PTEN) loss, as well as aberrant activation of the PI3K/AKT and RAS/MAPK pathways drive sustained PD-L1 upregulation across cancer types [53].

Extensive post-translational modifications and intracellular trafficking dynamically regulate PD-L1 abundance and function, determining its stability, subcellular localization, and signaling competence. At the post-translational level, glycosylation of PD-L1 stabilizes the protein and enhances its interaction with PD-1 [54] supporting its canonical immune function. In contrast, PD-L1 acetylation negatively regulates its trafficking to the plasma membrane, thereby attenuating PD-1/PD-L1 signaling. Notably, in human TNBC, reduced levels of glycosylated PD-L1 were observed relative to total PD-L1, concomitant with increased nuclear localization of PD-L1 [55].

EMT-inducing signals can also promote PD-L1 expression. TGF-β, a central regulator of EMT, upregulates PD-L1 expression in breast cancer cells through PI3K/AKT and MEK/ERK signaling; notably, this induction is a consequence of activation of the EMT program rather than a direct effect of TGF-β stimulation [50].

Other canonical oncogenic alterations, such as suppression of p53 signaling, activation of EMT, and pluripotency transcription factors (OCT4, SRY-box transcription factor 2 [SOX2], MYC proto-oncogene [MYC], ZEB1), are frequently accompanied by elevated PD-L1 expression [56], positioning PD-L1 as a downstream effector associated with tumor dedifferentiation programs. Accordingly, CD44, a stem cell biomarker associated with tumor initiation and metastasis [57], is also associated with PD-L1 regulation. In breast and lung cancer models, CD44 functions as an upstream regulator of PD-L1 [58].

Together, these findings support a model in which PD-L1 acts as a tumor-intrinsic signaling hub, integrating post-translational regulation, intracellular trafficking, transcriptional control, and interactions with stemness and EMT to promote tumor survival, plasticity, invasion, and therapeutic resistance beyond its canonical function. Central to these non-canonical functions is the convergence of PD-L1 signaling on metabolic and growth-regulatory pathways, particularly the mTOR axis. Through reciprocal interactions with PI3K/AKT, MEK/ERK, and mTORC1 signaling, PD-L1 seems to both respond to and reinforce oncogenic and microenvironmental signals, stabilizing proliferative, metabolic, and mesenchymal states.

## mTOR as a regulator of tumor plasticity

Tumor plasticity refers to the ability of malignant cells to dynamically reconfigure their molecular identity and phenotypic state in response to microenvironmental pressures, hypoxia, inflammation, or therapeutic interventions. Accordingly, it constitutes a central driver of tumor progression and therapeutic resistance [59]. Among the mechanisms underlying this plasticity, EMT stands out as a well-established program that enhances cellular motility, invasiveness, and phenotypic adaptability [60, 61]. Importantly, EMT has also been associated with the emergence of CSC-like subpopulations, thereby contributing to resistance to chemotherapy and radiotherapy [61, 62].

The PI3K/AKT/mTOR signaling pathway integrates metabolic and growth signals that regulate EMT and tumor plasticity. Under conditions of nutrient abundance, activation of this pathway sustains an anabolic state that promotes protein synthesis, mitochondrial biogenesis, and increased bioenergetic capacity, often associated with a predominance of oxidative phosphorylation (OXPHOS) [63]. In contrast, under hypoxia or metabolic stress, reduced mTOR activity allows activation of AMP-activated protein kinase (AMPK), shifting cellular metabolism toward glycolysis, which is more compatible with phenotypic transition states and survival in hostile microenvironments. This metabolic flexibility enables tumor cells to dynamically transition between epithelial, mesenchymal, and hybrid phenotypic states.

Multiple lines of evidence show that mTOR activation, particularly via mTORC1, promotes EMT by upregulating Snail and Twist and downregulating E-cadherin, thereby enhancing cellular motility and invasion [60, 64]. In colorectal tumors, inhibition of mTOR signaling reduces the expression of EMT-associated genes and significantly decreases cell migration and invasion [65]. In parallel, mTORC2 reinforces stemness properties by sustaining AKT signaling and other survival pathways that support self-renewal and the emergence of therapy-resistant subpopulations. Consequently, EMT, metabolic reprogramming, and CSC maintenance become interconnected processes that are directly or indirectly coordinated by mTOR signaling.

## The PD-L1/mTOR crosstalk: tumor migration and invasion

EMT-associated invasive phenotypes are characterized by N-cadherin and vimentin upregulation and E-cadherin loss. While EMT is primarily driven by transcription factors such as ZEB1, SNAIL1, and SNAIL2, its maintenance appears to be in part dependent on PD-L1, which contributes to tumor cell migration and sustains the mesenchymal state. This effect involves its crosstalk with the mTOR pathway and its upstream and downstream effectors, such as EGFR and MEK/ERK, which connects PD-L1 expression to cancer cells’ invasive capacity, reinforcing EMT alongside mTOR activation.

### PD-L1 relationship with epithelial-mesenchymal transition

Experimental data from PD-L1 overexpression, silencing and knockout models across multiple cell lines consistently support its role in cell migration and EMT. In gastric cancer cells, PD-L1 siRNA transfection led to a significant decrease in cell proliferation, migration and invasion [28]. A similar effect was observed in GBM cells, in which PD-L1 knockout severely impaired cell migration and invasion capacity [66].

In colorectal tumor cells, PD-L1 deletion or silencing impairs migration and invasion. In this model, the effect was dependent on PD-L1 isoform c, since its overexpression increased migration and invasion. In addition, isoform c increases EMT markers such as discoidin domain receptor 1 (DDR1) and fibronectin while reducing E-cadherin levels [67]. This shift reflects the hallmark EMT transition from cell–cell adhesion to cell–extracellular matrix (ECM) interactions [68, 69]. Accordingly, DDR1 has been associated with ECM remodeling by tumors [70].

These findings support a close link between PD-L1 expression and invasive capacity across cancer cells, inspiring studies on EMT markers under PD-L1 regulation. In lung cancer cells, transcription factor ZEB1 was reported to induce PD-L1 expression via miRNA-200 and IFN-γ signaling [71]. Consistently, metastatic renal carcinoma tissue shows PD-L1 overexpression [43], supporting the association between EMT-driven invasive phenotypes and PD-L1 expression.

In addition, the relationship between PD-L1 and EMT seems to be bidirectional: EMT-associated transcription factors can induce PD-L1 expression, while PD-L1 contributes to EMT maintenance. In breast cancer, EMT induction increases PD-L1, whereas PD-L1 silencing reverses EMT markers, with a positive correlation between EMT gene signatures and PD-L1 expression in patient samples [50]. Similarly, in NSCLC, TNF-α and TGF-β co-stimulation elevates PD-L1 via ZEB1 and SNAIL1, linking inflammatory signaling to EMT and PD-L1 upregulation [72]. The findings regarding PD-L1 and EMT are summarized in Table 1.

This data indicates that PD-L1 may not be merely a passive biomarker of EMT-associated processes. It could rather be an active player sustaining this invasive phenotype while also being induced by it. In this context, signaling pathways associated with PD-L1 regulation, as well as PD-L1 itself, may represent promising targets for tumor metastasis therapy.

**Table 1:** PD-L1 manipulation and its effects on EMT and invasive phenotype across cancer models

Study cancer type and model	PD-L1 manipulation	Pathway involved	Phenotypic outcome	References
Gastric cancer cell line	siRNA knockdown	–	↓ proliferation, migration, invasion	[28]
GBM cell lines	CRISPR knockout	–	↓ migration and invasion capacity	[66]
Colorectal tumor cell lines	Deletion/silencing; isoform c overexpression	–	Loss: ↓ migration/invasion. Isoform c overexpression: ↑ migration/invasion, ↑ DDR1 and fibronectin, ↓ E-cadherin	[67]
Lung cancer cell lines/mouse model/patient tissue	Endogenous (ZEB1-driven induction)	miR-200/IFN-γ → ZEB1	ZEB1 induces PD-L1 expression	[71]
Metastatic renal cell carcinoma patient tissue/cell lines	Knockdown Overexpression	SREBP-1c	PD-L1 overexpression associated with EMT-driven phenotype	[43]
Breast cancer (claudin-low) patient tissue	Silencing; EMT induction	PI3K/AKT, MEK/ERK (TGF-β-driven)	EMT induction ↑ PD-L1; PD-L1 silencing reverses EMT markers; positive correlation between EMT signature and PD-L1 in patient samples	[50]
NSCLC cell lines	Endogenous (TNF-α + TGF-β co-stimulation)	ZEB1, SNAIL1	↑ PD-L1 via ZEB1/SNAIL1	[72]

Abbreviations: AKT, protein kinase B; CRISPR, clustered regularly interspaced short palindromic repeats; DDR1, discoidin domain receptor 1; EMT, epithelial–mesenchymal transition; ERK, extracellular signal-regulated kinase; GBM, glioblastoma; IFN-γ, interferon gamma; MEK, mitogen-activated protein kinase kinase; miR-200, microRNA-200; NSCLC, non-small cell lung cancer; PD-L1, programmed death-ligand 1; PI3K, phosphoinositide 3-kinase; siRNA, small interfering RNA; SNAIL1, Snail family transcriptional repressor 1; SREBP-1c, sterol regulatory element-binding protein 1c; TGF-β, transforming growth factor beta; TNF-α, tumor necrosis factor alpha; ZEB1, zinc finger E-box-binding homeobox 1; ↑, increase; ↓, decrease

### Reciprocal relationship of PD-L1 with the mTOR pathway and its upstream and downstream effectors

Since the suppression of PD-L1 can play a role in reversing EMT markers, it is plausible that signaling pathways modulating its expression can have an important role in maintaining this invasive phenotype. Accordingly, several studies reported that mTOR and other upstream factors that lead to its activation can be involved in the induction of PD-L1 expression.

In a mouse model of lung squamous carcinoma with biallelic deletion of liver kinase B1 (Lkb1) and PTEN, both negative regulators of mTOR, PD-L1 levels were increased in lung tissue [73]. Similarly, NSCLC cell lines treated with PI3K and AKT inhibitors show reduced PD-L1 expression. In this model, the effect was mediated by mTORC1 rather than mTORC2, as regulatory-associated protein of mTOR (RAPTOR), but not rapamycin-insensitive companion of mTOR (RICTOR) silencing prevented PD-L1 expression. The same study also reported that mTOR activation through epidermal growth factor (EGF) and IFN-γ increased PD-L1 levels in NSCLC cell lines [74]. These data support a direct link between mTOR activity and PD-L1 regulation, whereby mTOR activation increases PD-L1 expression and may contribute to the EMT phenotype in cancer cells.

A comprehensive study evaluating PD-L1 overexpression demonstrated its impact on gene expression, cell migration, signaling pathways, and EMT markers in vitro and in vivo. In GBM cells overexpressing PD-L1, mRNA sequencing showed a differential expression of genes related to focal adhesion, ECM-receptor interaction and actin cytoskeleton regulation. It was also observed that PD-L1 overexpression increased cell migration, whereas its silencing reduced it. In this model, PD-L1 overexpression led to an increase in phosphorylated (p-)ERK1/2, but not p-AKT or p-glycogen synthase kinase 3 beta (p-GSK-3β); accordingly, an immunoprecipitation assay showed PD-L1 binding to RAS [42].

In this model, MEK inhibition reduced EMT markers SLUG and vimentin and increased E-cadherin, indicating that the RAS/MEK/ERK pathway mediates PD-L1-driven cell migration. The same study also observed that in vivo, GBM cells overexpressing PD-L1 showed greater tissue invasion than control cells. Moreover, the previously observed EMT markers were also observed in vivo [42]. Given that the MEK/ERK pathway induces mTOR activation through tuberous sclerosis complex 2 (TSC2) inhibition, PD-L1 binding to RAS may point to a direct link between tumor cells expressing PD-L1 and mTOR activation.

The upstream signaling pathways that lead to mTOR activation and consequent PD-L1 upregulation in tumor cells may be variable. In NSCLC, studies show that EGFR plays an important role in this effect. It was reported that cells from patients with stage IA-IIIA NSCLC showed a positive correlation between EGFR and PD-L1 expression. Moreover, EGF stimulation upregulated PD-L1 in lung adenocarcinoma cells, an effect abrogated by EGFR silencing. The exposure of these cells to PI3K/AKT and Janus kinase (JAK)/signal transducer and activator of transcription (STAT) inhibitors prevented EGF-induced PD-L1 expression [75]. Interestingly, another study in NSCLC and bronchial epithelial cells reported that PD-L1 expression was shown to depend on ERK1/2/p-c-Jun signaling, but not on the p-AKT/p-S6K pathway [76].

Besides EGFR signaling, it was also reported that NSCLC cell lines may upregulate PD-L1 upon anaplastic lymphoma kinase (ALK) rearrangements. ALK is a tyrosine kinase receptor and a member of the insulin receptor superfamily. In NSCLC, ALK activation commonly occurs through fusion with echinoderm microtubule-associated protein-like 4 (EML4), generating a constitutively active tyrosine kinase [77]. Cells harboring EML4–ALK rearrangements exhibit increased PD-L1 expression, which is attenuated by ALK inhibition or silencing. In this context, pharmacological inhibition of MEK/ERK and PI3K pathways also reduced PD-L1 levels, supporting the involvement of canonical downstream signaling cascades. Consistently, PD-L1 expression was positively correlated with EML4–ALK rearrangement in tumor biopsy samples [78].

In parallel, evidence from prostate cancer models suggests that PD-L1 regulation may also be influenced by stress-responsive pathways. In prostate cancer cells, PD-L1 expression was shown to depend on ataxia telangiectasia mutated (ATM) kinase via the JAK/STAT pathway. ATM silencing reduced PD-L1 expression and impaired cell migration, accompanied by decreased phosphorylation of JAK1, JAK2, and STAT3. Interestingly, JAK inhibition, but not STAT inhibition, was sufficient to downregulate PD-L1 and reverse EMT markers [79]. Although ATM is classically associated with DNA damage responses, it has also been implicated in cell migration through modulation of CD13 and matrix metalloproteinases (MMPs) such as MMP-2 and MMP-9 [80]. Together, these findings raise the possibility that distinct oncogenic and stress-related pathways may converge on PD-L1 regulation through partially overlapping downstream networks, potentially involving PI3K/AKT/mTOR and JAK/STAT axes, and thereby shaping both immune evasion and migratory phenotypes.

Downstream of mTOR, multiple transcription factors regulate PD-L1 expression. In EGFR-mutant NSCLC cell lines, gefitinib reduced IL-6, PD-L1 and p-STAT3. Accordingly, STAT3 silencing also reduced PD-L1 levels [81]. Transcription factor MYC, an oncogene associated with many tumor types, has also been shown to play a role in PD-L1 expression. The overexpression of bridging integrator 1 (BIN1), a MYC adaptor with tumor suppressor activity, downregulated PD-L1 mRNA and protein levels in H1975 and HCC827 cell lines, while BIN1 silencing led to increased PD-L1 in H460 and H1299 cell lines [82]. Under this regulatory network, mTOR acts as a key regulator of PD-L1 by influencing factors like STAT3 and MYC, linking metabolism to immune evasion and tumor invasion. These findings about PD-L1/mTOR crosstalk are summarized in Table 2.

**Fig. 1:**
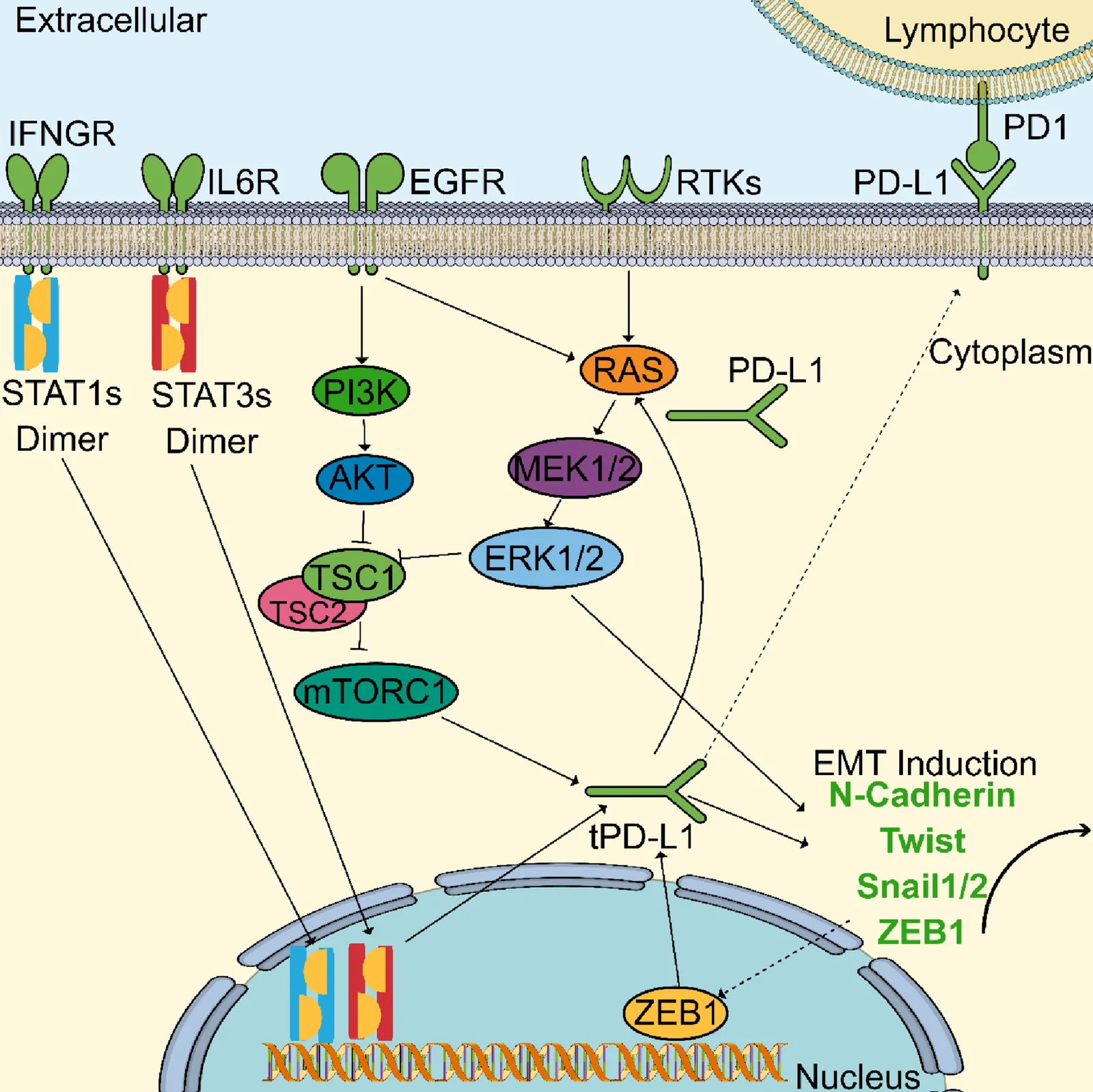
PD-L1/mTOR crosstalk in EMT and tumor cell invasiveness. Beyond its immune checkpoint role by binding to lymphocyte PD-1, PD-L1 induces mTOR activity through RAS binding and sustains a mesenchymal phenotype by inducing and maintaining EMT. Abbreviations: AKT, protein kinase B; EGFR, epidermal growth factor receptor; EMT, epithelial–mesenchymal transition; ERK1/2, extracellular signal-regulated kinases 1 and 2; IFNGR, interferon gamma receptor; IL6R, interleukin 6 receptor; MEK1/2, mitogen-activated protein kinase kinases 1 and 2; mTOR, mechanistic target of rapamycin; mTORC1, mTOR complex 1; PD1 (PD-1), programmed cell death protein 1; PD-L1, programmed death-ligand 1; t-PD-L1, transduced PD-L1; PI3K, phosphoinositide 3-kinase; RAS, rat sarcoma viral oncogene homolog; RTKs, receptor tyrosine kinases; Snail1/2, Snail family transcriptional repressors 1 and 2; STAT1, signal transducer and activator of transcription 1; STAT3, signal transducer and activator of transcription 3; TSC1, tuberous sclerosis complex 1; TSC2, tuberous sclerosis complex 2; ZEB1, zinc finger E-box-binding homeobox 1. Original figure created by the authors using Mind the Graph (www.mindthegraph.com)

Collectively, this data indicates that while mTOR plays a key role in PD-L1 upregulation, its upstream signaling varies across tumor types. Moreover, EMT-driven PD-L1 expression may also activate mTOR, creating a feedback loop that sustains both PD-L1 expression and the EMT phenotype, as summarized in Fig. 1. Therefore, identifying tumor-specific upstream regulators of mTOR is essential to guide combination therapies that combine mTOR inhibitors with agents targeting these pathways.

**Table 2:** Upstream regulators of mTOR/PD-L1 signaling and their reported effects on PD-L1 expression

Study cancer type and model	Upstream manipulation	Pathway involved	Effect on PD-L1 / phenotype	References
Lung squamous cell carcinoma mouse model/patient tissue	Biallelic Lkb1 + PTEN deletion	mTOR (loss of negative regulation)	↑ PD-L1 in lung tissue	[82]
NSCLC cell lines/mouse model	PI3K/AKT inhibitors; RAPTOR vs. RICTOR silencing; EGF/IFN-γ stimulation	PI3K/AKT/mTORC1	PI3K/AKT inhibition ↓ PD-L1 (mTORC1-dependent, RAPTOR not RICTOR); EGF/IFN-γ ↑ PD-L1	[74]
GBM cell lines	PD-L1 overexpression/silencing	RAS/MEK/ERK → TSC2 inhibition → mTOR	Overexpression ↑ p-ERK1/2 (not p-AKT/p-GSK-3β); PD-L1 binds RAS; ↑ migration/invasion in vitro and in vivo; MEK inhibition reverses EMT markers	[42]
NSCLC (patient samples/lung adenocarcinoma cell lines)	EGFR silencing; EGF stimulation; PI3K/AKT and JAK/STAT inhibitors	EGFR → PI3K/AKT, JAK/STAT	Positive EGFR/PD-L1 correlation (stage IA–IIIA); EGF ↑ PD-L1, abrogated by EGFR silencing or PI3K/AKT/JAK/STAT inhibition	[75]
NSCLC and bronchial epithelial cell lines	Pathway-dependency testing (no direct manipulation stated)	ERK1/2/p-c-Jun (not p-AKT/p-S6K)	PD-L1 expression depends on ERK1/2/p-c-Jun, independent of AKT/S6K	[76]
NSCLC EML4–ALK⁺ cell lines/ patient tissue	ALK inhibition/silencing; MEK/ERK and PI3K inhibition	ALK → MEK/ERK, PI3K	EML4–ALK rearrangement ↑ PD-L1; ALK inhibition/silencing ↓ PD-L1; positive correlation in biopsies	[77, 78]
Prostate cancer cells cell lines/patient tissue	ATM silencing; JAK vs. STAT inhibition	ATM → JAK/STAT	ATM silencing ↓ PD-L1, ↓ migration, ↓ p-JAK1/2/p-STAT3; JAK (not STAT) inhibition ↓ PD-L1 and reverses EMT markers	[79]
EGFR-mutant NSCLC cell lines	Gefitinib; STAT3 silencing	EGFR → IL-6 → STAT3	Gefitinib ↓ IL-6, PD-L1, p-STAT3; STAT3 silencing ↓ PD-L1	[81]
NSCLC cell lines/patient tissue	BIN1 overexpression vs. silencing	MYC/EGFR-MAPK (BIN1 as MYC adaptor)	Overexpression ↓ PD-L1 (H1975, HCC827); silencing ↑ PD-L1 (H460, H1299)	[82]

Abbreviations: AKT, protein kinase B; ALK, anaplastic lymphoma kinase; ALK⁺, ALK rearrangement-positive; ATM, ataxia telangiectasia mutated; BIN1, bridging integrator 1; CD274, gene encoding PD-L1; EGF, epidermal growth factor; EGFR, epidermal growth factor receptor; EML4, echinoderm microtubule-associated protein-like 4; EMT, epithelial–mesenchymal transition; ERK, extracellular signal-regulated kinase; GBM, glioblastoma; GSK-3β, glycogen synthase kinase 3 beta; IFN-γ, interferon gamma; IL-6, interleukin 6; JAK, Janus kinase; Lkb1, liver kinase B1; MAPK, mitogen-activated protein kinase; MEK, mitogen-activated protein kinase kinase; mTOR, mechanistic target of rapamycin; mTORC1, mTOR complex 1; MYC, MYC proto-oncogene; NSCLC, non-small cell lung cancer; p-, phosphorylated; PD-L1, programmed death-ligand 1; PI3K, phosphoinositide 3-kinase; PTEN, phosphatase and tensin homolog; RAPTOR, regulatory-associated protein of mTOR; RAS, rat sarcoma viral oncogene homolog; RICTOR, rapamycin-insensitive companion of mTOR; S6K, ribosomal protein S6 kinase; STAT, signal transducer and activator of transcription; STAT3, signal transducer and activator of transcription 3; TSC2, tuberous sclerosis complex 2; ↑, increase; ↓, decrease

## Therapeutic implications and perspectives

Most, if not all, therapies targeting PD-L1 aim primarily to block its immune checkpoint function, using antibodies that recognize its extracellular domain or its receptor. However, the data discussed in this review proposes a view in which the goal of targeting PD-L1 involves inhibiting its intracellular tumor signaling along with mTOR pathway inhibition to reduce tumor metastatic potential.

### Preclinical evidence

Although many studies have investigated the importance of both PD-L1 and mTOR in tumor migration and EMT, thus pointing to a strong mechanistic rationale for their combined role, there is still a lack in the literature of direct preclinical evidence for the synergistic effect of combined PD-L1 and mTOR pathway inhibition on tumor migration. In SW480 and SW480R colorectal cancer cell lines, it was reported that the combination of PD-L1 and mTOR small molecule inhibitors reduced cell migration [83]. In this study, the effect was dependent on concomitant PD-L1 and PI3K/mTOR inhibition by BMS-1166 and BEZ235, respectively.

To our knowledge, this is the only study that evaluated cancer cell migration with both PD-L1 and mTOR inhibition. Further studies are needed to evaluate whether this type of combination therapy is relevant across different cancer cell types, consistent with the mechanistic data reported in isolated models.

### Clinical evidence

Most clinical trials addressing PD-L1 combination therapy do not directly investigate mTOR inhibition and instead focus on TKIs with different targets upstream of the mTOR pathway. Nonetheless, some of these studies may prove useful for our discussion and are thus listed below.

### Ongoing clinical trials associating PD-L1 and mTOR pathway inhibitors

COSMIC-021, a phase 1b open-label study (NCT03170960) aims to assess the safety, tolerability and preliminary efficacy of cabozantinib in association with atezolizumab in cohorts of different solid tumors. Cabozantinib is a multi-target TKI, whose main targets are mesenchymal-epithelial transition factor (c-MET), vascular endothelial growth factor receptor (VEGFR) isoforms, and AXL receptor tyrosine kinase (AXL). Published data so far shows promising efficacy results for further clinical trials [84–87].

A phase II trial for advanced-stage kidney cancer (NCT04981509) aims to evaluate the objective response for the combination of the anti-PD-L1 and anti-vascular endothelial growth factor (VEGF) monoclonal antibodies atezolizumab and bevacizumab, respectively, with the EGFR inhibitor erlotinib. Since this is a phase II trial, the main goal of the study is to evaluate the incidence of adverse events. Its completion is estimated for 2026-09-11, but no results have been published so far.

It is important to note that these clinical trials do not have the specific goal of evaluating tumor metastasis, and their designs were not intended to isolate PD-L1/mTOR axis inhibition specifically. However, the safety and efficacy data obtained from these trials may pave the way for new studies aiming for this combination therapy.

### Completed clinical trials associating PD-L1 and mTOR pathway inhibitors

Besides ongoing studies, there are published data from previous trials that investigated the therapeutic efficacy of immune checkpoint inhibitors (ICIs) combined with inhibitors of mTOR upstream receptors.

#### Anti-PD-L1 and AKT inhibitors

The direct association of the AKT inhibitor ipatasertib with the anti-PD-L1 monoclonal antibody atezolizumab was studied in ICE-CAP (NCT03673787), a single-center phase I trial. Published data so far report results from three cohorts: solid tumor patients (cohort A1) and two cohorts of GBM patients (the relapsed GBM cohort A2 and the exploratory cohort B3). Although it was conducted in a small number of patients and its main goal was to determine the maximum tolerated dose and recommended phase II dose, the study reported interesting results from preliminary efficacy and pharmacodynamics of this therapy.

The administration of combined ipatasertib with atezolizumab was shown to be clinically well tolerated. It was reported that among responsive patients, a possible mechanism by which ipatasertib acted was in an immunomodulatory context. In biopsy samples, it was observed a reduction in immunosuppressive regulatory T cells (Tregs) and an increase in CD8^+^ T lymphocytes followed by ipatasertib administration. Unfortunately, due to limited biopsy tissue availability, AKT pathway inhibition was not analyzed. In the GBM cohort, some of the responsive patients had a historic of recurrent GBM, a clinical profile associated with poor immunotherapy response [88]. It may be the case that AKT inhibition played a role in overcoming resistance. However, there is a clear need for future trials to evaluate this specific population of patients regarding anti-PD-L1 therapy associated with AKT inhibitors.

Since AKT inhibition by ipatasertib is not tissue-specific, both tumor-intrinsic and immune-cell inhibition may occur in parallel, and anti-PD-L1 effects may likewise occur both by inhibiting its immune checkpoint role and by modulating tumor-intrinsic signaling. Notably, the observed immunomodulatory effect—Treg depletion and increased CD8⁺ T cell infiltration—occurred independently of PI3K mutational status or PTEN loss, suggesting that it may be predominantly driven by AKT inhibition within immune cells themselves, rather than depending on pathway hyperactivation in the tumor. This inference remains indirect, however, since the absence of a genotype-dependent difference does not exclude a smaller, less detectable tumor-intrinsic contribution. Future studies incorporating cell-type-resolved biopsy analysis—for instance, through single-cell or spatial profiling—would be needed to disentangle these contributions and clarify which cells and pathways drive the therapeutic effect of this combination.

#### Anti-PD-L1 and tyrosine kinase inhibitors

Besides direct mTOR inhibition, clinical trials have also been carried out targeting both PD-L1 and tyrosine kinase receptors, such as VEGFR and c-MET. Since these tyrosine kinase receptors are upstream activators of mTOR [89, 90], along with other associated signaling pathways, their association with anti-PD-L1 therapy in clinical trials will be discussed.

The phase III JAVELIN Renal 101 (NCT02684006) trial compared the association of Avelumab, an anti-PD-L1 antibody, in association with axitinib, a VEGFR inhibitor, in comparison to sunitinib, a multi-target TKI, including VEGFR. In the overall population, avelumab plus axitinib versus sunitinib showed an overall survival (OS) of 44.8 vs. 38.9 months, which did not reach statistical significance; in contrast, both the 5-year progression-free survival (PFS) (12.0% vs. 4.4%) and objective response rate (ORR) (59.7% vs. 32.0%) were significantly improved with the combination [91].

A post-hoc analysis showed that among advanced renal cell carcinoma (RCC) patients whose tumor presented sarcomatoid histology, which is associated with a poorest prognosis, the ORR was higher in the avelumab plus axitinib group (46.8%) compared with the sunitinib group (21.3%), and PFS was reported to be 7 and 4 months for these groups, respectively [92], consistent with the overall results.

Sarcomatoid RCC is a histological subtype associated with EMT markers [93] and higher levels of PD-L1 when compared to other RCC [94]. The data from this trial indicates that patients whose tumors have this phenotype may benefit from the combination of anti-PD-L1 and TKIs. However, it is not possible to conclude that the observed ORR and PFS effect was dependent on PD-L1/mTOR axis inhibition rather than an increased immune response. A better understanding of this combination therapy’s pharmacodynamics would be possible by investigating signaling pathways and immune infiltration in tissue samples.

Interestingly, the combination of cabozantinib and atezolizumab was also studied in CONTACT-03 (NCT04338269), a phase III trial in patients with RCC after progression with prior ICI treatment. The authors concluded that there was no improvement in outcomes with this combination and that it led to increased toxicity [95]. Although the JAVELIN Renal 101 and CONTACT-03 trials presented a similar pharmacodynamic strategy, the reported results are, in part, contradictory. A plausible explanation for this may lie in the selected patient population: CONTACT-03 trial was conducted in patients that had already undergone ICI therapy. This indicates that prior ICI treatment may influence the therapeutic efficacy of combined PD-L1 and TKIs.

In another study, the safety and efficacy of the c-MET inhibitor savolitinib combined with durvalumab were investigated in metastatic papillary renal cancer in the CALYPSO phase II trial. c-MET is a tyrosine kinase receptor for hepatocyte growth factor (HGF) associated with cell stemness, invasiveness and metabolic reprogramming. When activated, it is responsible for signal transduction through multiple pathways, including STAT3 phosphorylation and PI3K/AKT/mTOR. Also, c-MET activation can occur through a non-canonical mechanism via the SLUG transcription factor [96].

This study reported that ORR for the intention-to-treat (ITT) population and c-MET-driven patients was 34% and 53%, respectively. Accordingly, the reported PFS was 6.5 months for the ITT population and 13.9 months for c-MET-driven patients, while OS was 18.3 vs. 27.4 months for these groups, respectively. Interestingly, PD-L1 and tumor mutation burden did not correlate with the observed response [97]. These data indicate that combining anti–PD-L1 with upstream mTOR inhibition may have clinical relevance for targeting pathways linked to tumor stemness and metastasis, especially targeting mTOR upstream pathways already known to drive a specific tumor type, such as c-MET in this cohort. The preliminary data from this phase II trial encourages a phase III investigation to further confirm the efficacy of this therapeutic option.

### Small-molecule PD-L1 inhibitors

Although the canonical functions of the PD-L1 axis depend on the interaction of its extracellular domain with PD-1 on lymphocytes, the inhibition of its non-canonical signaling may benefit from small-molecule PD-L1 inhibitors by targeting its intracellular form. In this context, small-molecule inhibitors have been studied for targeting the PD-L1/PD-1 interaction, but also for suppressing PD-L1 expression, promoting its degradation, targeting its epigenetic regulation and transcriptional pathways associated with its expression [98, 99]. This new approach to targeting PD-L1’s intracellular functions may provide a future alternative for targeting this axis.

## Conclusion

This review focused on PD-L1’s non-canonical functions, especially its role in cancer cell migration and its intersection with upstream and downstream effectors of the mTOR pathway. Cumulative preclinical evidence positions PD-L1 as a central regulator of EMT, acting both downstream of EMT-inducing signals and as a driver and regulator of invasive phenotype across cancers. Intracellularly, this function is coupled with mTOR activity, forming a reciprocal signaling hub in which PD-L1 and mTOR sustain EMT-associated programs.

The presented evidence permits us to conclude that (1) PD-L1 upregulation is closely associated with EMT phenotype in an active manner across different cancer types; (2) the mTOR pathway may have a relevant role in PD-L1 expression and thus be associated with EMT induction and maintenance and (3) PD-L1 itself may sustain mTOR signaling.

However, it is important to note this review’s limitations. As a narrative rather than a systematic review, there were no specific criteria for study selection or exclusion, and it was rather selected by reported experimental data on the proposed signaling pathways in different cancer models. This may lead to a conceptual framework that only partially represents the PD-L1/mTOR intersection in tumor migration, and future work should aim to elucidate the context-specific relevance of this axis.

### Future directions

The cumulative data on PD-L1 functions in cancer cells permits us to think of targeting it to hinder cancer progression through different mechanisms, especially those associated with EMT. It is necessary, therefore, to better understand its signaling hub in addition to its immune-related functions. Important questions to be addressed are (1) which PD-L1 domains are associated with intracellular signaling; (2) what are the mechanisms by which PD-L1 sustains EMT; and (3) in which contexts it promotes mTOR activation. A better understanding of these mechanisms will allow for better drug design for PD-L1 inhibitors beyond targeting its membrane-bound form.

Regarding mTOR pathway inhibition, it is relevant to understand which proteins are associated with its upregulation across different cancer types. Defining the context-specific upstream regulators of mTOR will be critical to stratify patients and refine combination strategies targeting this axis.

Beyond addressing these mechanistic questions, direct preclinical evidence evaluating the effects of combined PD-L1 and mTOR inhibition on cell migration, invasion, and EMT is still needed to establish their synergistic relevance. Such evidence would provide a rational basis for designing combination strategies that target both the immune and tumor-intrinsic functions of PD-L1, potentially expanding the therapeutic reach of PD-L1-directed therapies beyond immune checkpoint blockade alone.

## Data Availability

No datasets were generated or analysed during the current study.

## Abbreviations

AKT: Protein kinase B
ALK: Anaplastic lymphoma kinase
AMPK: AMP–activated protein kinase
ATM: Ataxia telangiectasia mutated
AXL: AXL receptor tyrosine kinase
BIN1: Bridging integrator 1
BMI1: BMI1 proto–oncogene, polycomb ring finger
c-MET: Mesenchymal–epithelial transition factor
CSC: Cancer stem cell
CTNNB1: Catenin beta 1
DDR1: Discoidin domain receptor 1
ECM: Extracellular matrix
EGF: Epidermal growth factor
EGFR: Epidermal growth factor receptor
EML4: Echinoderm microtubule–associated protein–like 4
EMT: Epithelial–mesenchymal transition
ERK: Extracellular signal–regulated kinase
GAS6: Growth arrest–specific 6
GBM: Glioblastoma
GSK: 3β–Glycogen synthase kinase 3 beta
HGF: Hepatocyte growth factor
HIF: Hypoxia–inducible factor
HMGA1: High mobility group AT–hook 1
ICI: Immune checkpoint inhibitor
IFN-γ: Interferon gamma
IgC: Immunoglobulin constant
IgV: Immunoglobulin variable
IL-6: Interleukin 6
IL-17: Interleukin 17
ITIM: Immunoreceptor tyrosine–based inhibitory motif
ITSM: Immunoreceptor tyrosine–based switch motif
ITT: Intention–to–treat
JAK: Janus kinase
KPNB1: Karyopherin beta 1
LKB1: Liver kinase B1
MAPK: Mitogen–activated protein kinase
MEK: Mitogen–activated protein kinase kinase
MMP: Matrix metalloproteinase
mTOR: Mechanistic target of rapamycin
mTORC1: Mechanistic target of rapamycin complex 1
mTORC2: Mechanistic target of rapamycin complex 2
mTORC3: Mechanistic target of rapamycin complex 3
MYC: MYC proto–oncogene
NANOG: Nanog homeobox
NSCLC: Non–small cell lung cancer
OCT4: Octamer–binding transcription factor 4
ORR: Objective response rate
OS: Overall survival
OXPHOS: Oxidative phosphorylation
p70S6K: phosphorylated 70 kDa ribosomal protein S6 kinase
PD-1: Programmed cell death protein 1
PD-L1: Programmed death–ligand 1
PD-L2: Programmed death–ligand 2
PFS: Progression–free survival
PI3K: Phosphoinositide 3–kinase
PTEN: Phosphatase and tensin homolog
RAPTOR: Regulatory–associated protein of mTOR
RAS: Rat sarcoma viral oncogene homolog
RCC: Renal cell carcinoma
RICTOR: Rapamycin–insensitive companion of mTOR
S6K: Ribosomal protein S6 kinase
SHP-1: Src homology region 2 domain–containing phosphatase 1
SHP-2: Src homology region 2 domain–containing phosphatase 2
SLUG: Snail family transcriptional repressor 2 (synonym of SNAIL2)
SNAIL1: Snail family transcriptional repressor 1
SNAIL2: Snail family transcriptional repressor 2
SOX2: SRY–box transcription factor 2
SREBP-1: Sterol regulatory element–binding protein 1
STAT: Signal transducer and activator of transcription
STAT1: Signal transducer and activator of transcription 1
STAT3: Signal transducer and activator of transcription 3
TCR: T–cell receptor
TGF-β: Transforming growth factor beta
TKI: Tyrosine kinase inhibitor
TME: Tumor microenvironment
TNBC: Triple–negative breast cancer
TNF-α: Tumor necrosis factor alpha
Treg: Regulatory T cell
TSC2: Tuberous sclerosis complex 2
VEGF: Vascular endothelial growth factor
VEGFR: Vascular endothelial growth factor receptor
WIP: WAS/WASL–interacting protein
ZEB1: Zinc finger E–box–binding homeobox 1
